# Is It ‘Pseudoneurotic Psychosis’? Reporting Mystical Delusions in a Grieving Adolescent

**DOI:** 10.1192/j.eurpsy.2023.1566

**Published:** 2023-07-19

**Authors:** S. G. Rodrigues, M. F. Liz, M. E. Araújo

**Affiliations:** 1 Centro Hospitalar e Universitário do Porto, Porto, Portugal

## Abstract

**Introduction:**

The entity of ‘pseudoneurotic schizophrenia’ was coined in 1949 by Hoch and Polatin to define emerging psychotic symptoms, namely formal thought disorder and emotional dysregulation, in patients previously presenting with neurotic functioning. Although currently considered to be outdated, the term paved way for the concept of ‘borderline disorders’, known for their difficult assessment.

**Objectives:**

To highlight the obstacles in diagnosing clinical presentations of overlapping psychotic and neurotic symptomatology.

**Methods:**

We report a case of an adolescent admitted for presumed psychosis, later to display fast clinical improvement and significant neurotic personality traits.

**Results:**

A 17-year-old male with no previous psychiatric follow-up, except for brief psychotherapeutic intervention at the age of 11, following the death of his grandfather.

He presented with a sudden change in behavior and sleep since the week before, coincident with acknowledging the loss of his best friend in a car accident. Upon evaluation, he presented with unstable gait. He seemed fatigued but displayed inappropriate restricted affect. He reported perceiving bizarre, meaningful signs everywhere concerning his own death since the event. Additionally, he detailed feelings of lethargy and unexplained sadness, relying on the nihilistic delusional beliefs that he had been in deep sleep and he would die soon. At admission, he was prescribed with aripriprazol 5mg id.

Throughout his stay in the hospital, he maintained consistently adequate, calm behavior. During inpatient clinical interviews, he showed clear insight into the aforementioned behavior. He provided clear, logical information referring to his past grief process and remaining trauma, reporting coping mechanisms based on spiritual beliefs

Prescription medication was interrupted soon after admission, with no noticeable changes. At dismissal, despite remaining sad concerning the death of his friend, there was no signs of psychotic symptoms or other significant mental distress.

**Conclusions:**

In this report, we emphasize the hazards of differential diagnosis between psychosis and emotional dysregulation with underlying neurotic traits. There is conflicting evidence on the concept of ‘pseudoneurotic’ presentations, specifically ‘pseudoneurotic schizophrenia’. Available information on distinguishing between overlapping psychotic and neurotic features in adolescents is even more scarce. To perform extended, multidisciplinary evaluations might be key in accurately assessing these patients.

**Disclosure of Interest:**

None Declared

